# Effect of platelet-rich plasma versus steroid injection in plantar fasciitis: a randomized clinical trial

**DOI:** 10.1186/s12891-023-06277-1

**Published:** 2023-03-07

**Authors:** Rachit Sharma, Narendra Kumar Chaudhary, Mandeep Karki, Dev Ram Sunuwar, Devendra Raj Singh, Pranil Man Singh Pradhan, Prakash Gyawali, Sailendra Kumar Duwal Shrestha, Kailash Kumar Bhandari

**Affiliations:** 1 Nepal Orthopaedic Hospital, Kathmandu, Nepal; 2 Department of Nutrition and Dietetics, Nepal Armed Police Force Hospital, Kathmandu, Nepal; 3 School of Human and Health Sciences, University of Huddersfield, Huddersfield, UK; 4 Department of Community Medicine and Public Health, Institute of Medicine, Tribhuvan University, Tribhuvan, Nepal; 5 Department of Orthopaedics, Nepal Armed Police Force Hospital, Kathmandu, Nepal

**Keywords:** Plantar fascia, Plantar fasciitis, Platelet-rich plasma, Steroid

## Abstract

**Background:**

Plantar fasciitis (PF) is a common orthopaedic problem, with heel pain worsening the quality of life. Although steroid injection is often used if the conservative treatment fails, Platelet-Rich Plasma (PRP) injection is gaining popularity due to its safety and long-lasting effect. However, the effect of PRP versus steroid injection in PF has not been studied yet in Nepal. Therefore, this study aimed to assess the effect of PRP compared with steroid injection in the treatment of PF.

**Methods:**

This study was a single-center, hospital-based, open-label, parallel-group randomized clinical trial to compare the effect of PRP injection with steroid injection in plantar fasciitis between August 2020 and March 2022. A total of 90 randomly selected participants aged 18 to 60 years suffering from plantar fasciitis with failed conservative treatment were intervened. The American Orthopaedic Foot and Ankle Society (AOFAS) and the Visual Analog Scale (VAS) scoring system were used to evaluate functional mobility and pain before and after the intervention for three and six months, respectively. Statistical analyses were performed using a Student’s two-sample t-test. P-value < 0.05 was considered statistically significant.

**Results:**

The PRP injection showed a better outcome than the steroid injection in six months follow-up. The mean (± SD) VAS score was significantly decreased in the PRP group (1.97 + 1.13) than in the steroid group (2.71 ± 0.94) with the group difference of -0.73 (95% CI: -1.18 to -0.28) at six months. Similarly, there was a significant increase in the AOFAS scores in the PRP group (86.04 ± 7.45) compared to the steroid group (81.23 ± 9.60) at six months of follow-up with a group difference of 4.80 (95% CI: 1.15 to 8.45). There was also a significant reduction of plantar fascia thickness in the PRP group compared to that of the steroid group (3.53 ± 0.81 versus 4.58 ± 1.02) at six months of follow-up with the group difference of -1.04 (95% CI: -1.44 to -0.65).

**Conclusion:**

The PRP injection showed better outcomes than steroid injection in plantar fasciitis treatment over the course of six months. Further research with a larger population and longer follow-up than six months is needed to generalize the findings and their long-term efficacy.

**Trial registration:**

NCT04985396. First registered on 02 August 2021. (https://clinicaltrials.gov/ct2/show/NCT04985396)

**Supplementary Information:**

The online version contains supplementary material available at 10.1186/s12891-023-06277-1.

## Introduction

Plantar fasciitis (PF), better termed as plantar fasciosis [1, 2], is a degeneration of plantar fascia leading to an inflammatory reaction [3]. It occurs mostly due to the biomechanical stress on the plantar fascia [4]. The plantar fascia is a thin elastic fibrous band of connective tissue aligned in a longitudinal orientation with a rich extracellular matrix predominantly in the Hyaluronan [5]. Fasciacytes, a new cell found in the plantar fascia, first termed by Stecco et al., 2018 is devoted to the production of hyaluronan, which promotes the gliding function between the deep fascia and muscle [6]. Plantar fascia lies in close connectivity to the para tendon of Achilles through the heel periosteum. Therefore, any degenerative or inflammatory process within the para tendon of Achilles can hinder normal foot kinematics rendering plantar fascia thickness increment leading to plantar fasciitis [7].

The PF worsens the quality of life [8, 9] with a lifetime global prevalence of 10% [10], more common in females than males [11] due to the difference in lifestyle and health status between both sexes [12]. The prevalence of plantar fasciitis is 7.5% in the UK, 3.6% in Australia [13], 59% in India among the age group of 40 to 50 years [14], and 57.8% in Saudi Arabia [15]. Physicians have 8.14%, nurses have 13.11% [16], and athletes have 5 to 18% prevalence [13]. However, the prevalence of plantar fasciitis in Nepal is unknown.

PF resolves in 80–90% of cases within ten months [17–19]. Non-surgical treatments such as non-steroid anti-inflammatory drugs (NSAIDs), shoe inserts, stretching exercises, or extracorporeal shockwave therapy [20–23] are the first-line treatment of PF [24], successful in up to 90% of cases [25] and steroid as an injection (SI) therapy is traditionally practiced if non-operative treatment fails [26]. It is effective because of its anti-inflammatory properties, associated with the risk of plantar fascia rupture, heel fat pad atrophy [27], lateral plantar nerve injury secondary to injection, and calcaneal osteomyelitis and iontophoresis, burning of the underlying skin [28]. A steroid injection may offer short-term relief, but it did not find long-term benefit in six months follow-ups compared to a placebo in a recent review [29]. On the other hand, Platelet Rich Plasma (PRP) has strong anti-inflammatory properties with no adverse effects on the plantar fascia structure. It contains high levels of growth factors and anti-inflammatory cytokines, which potentially ameliorate degenerative conditions [30], prevent infections [31], and enhance wound healing, bone healing, and tendon healing [32]. Therefore, PRP has been a biological option in treating the PF [33].

PRP therapy is a relatively new approach in the regenerative medicine [34]. Nowadays, it has been a promising solution to many orthopaedic problems such as tendinopathies, non-union, and arthritis of the knee [35]. Of course, it has gained popularity in treating Achilles tendinopathies [36]. However, there is also controversy among orthopaedic surgeons about the effectiveness of the PRP [37]. The high cost of commercially available PRP kits demotivates the use of PRP therapy in most cases [38].

Furthermore, the PRP preparation in the laboratory is a lengthy process, taking approximately 35 to 40 min but yields an increase in initial platelet count from 104.47 to 196.82%, allowing excellent clinical application [39]. There is a paucity of evidence to compare the effect of PRP with steroid injection for the management of plantar fasciitis in poor resource countries like Nepal. As far as we know, there hasn’t been single research undertaken in Nepal to compare the efficacy of steroid injection and PRP. This study will be important in developing countries like Nepal from a different perspective. Developing countries have a high prevalence of barefoot activity [40], which leads to the anatomy of foot pronation [41] causing chronic PF [42], which accounts for about 70 − 86% of patients [20]. A large portion of the population (between 63 and 72% ) wears inappropriate shoes [43] leads to plantar fasciitis [44]. Similarly, an intra-articular or soft tissue steroid injection may lead to different health comorbidities [45]; one of them is the increase of acute coronary syndrome with seven folds [46], raising the medical cost as well as costs due to the loss of productivity [47]. On the other side, PRP treatment is low-cost, simple and minimally invasive in nature [48]. It is safer and more beneficial than steroid injection in PF [49, 50] and is considered superior to SI for long-term pain relief [51]. Different studies have also shown that the PRP treatment is an alternative to surgery [52–56], and data reveals that surgery may be required for approximately 5–10% of chronic plantar fasciitis [57]. As the handmade standard PRP is more reliable and cost-effective than commercially available PRP kits [38], the financial burden on the health care system can be minimized by establishing the standardized laboratory set-up and formulating the PRP preparation protocol in developing countries. Therefore, this study aimed to compare the effect of platelet-rich plasma injection with steroid injection for the treatment of plantar fasciitis in Nepal in terms of pain, functional mobility and also the change of plantar fascia thickness.

## Methods

### Study design and setting

The report of this trial followed the Consolidated Standards of Reporting Trials (CONSORT) 2010 updated guidelines for reporting randomized controlled trials [58]. This study was a single-centre, hospital-based, open-label, parallel-group randomized clinical trial to compare the effect of PRP injection with steroid injection in the treatment of Plantar fasciitis between August 2020 and March 2022. The study was conducted in the outpatient department of Nepal Orthopaedic Hospital, Kathmandu, Nepal. It is one of the Orthopaedic and Trauma care super-speciality centres in Nepal that provides only orthopaedic services. It is a 100 bedded charitable autonomous hospital run under the Nepal Disabled Association of Nepal with the support of the Patan Rotary club, Nepal, and different International Rotary Clubs [59]. The patients aged 18 to 60 years with a history of heel pain of more than six weeks with tenderness on palpation over medial calcaneum tuberosity and diagnosed as PF, those patients with failure of conservative treatment with physiotherapy, splints, and NSAIDs, those patients who were mentally fit, and those patients who provided written informed consent were included in the study. The patients with lumbar radiculopathy, existing trauma, previous surgery or any foot pathology, under aspirin treatment, bleeding disorders with low platelet counts, and systemic diseases like diabetes and rheumatoid arthritis were excluded.

### Sample size determination

The sample size was calculated based on a similar study conducted by Jain et al. (2015) in Wrightington Hospital, Wigan, UK, where the mean ± SD of the American Orthopaedic Foot and Ankle Society (AOFAS) score in both PRP Vs steroid groups were 88.50 ± 13.52 Vs 75.07 ± 20.13, respectively [60]. The sample size was calculated considering this data and taking a level of significance at 3% and power of 90%, using a test comparing independent two means in Stata/MP version 14.1 (StataCorp LP, College Station, Texas). The calculated sample size was 76. After adding a 20% dropout rate, the final sample size was 90 (45 participants in each group).

### Randomization

**Fig. 1:**
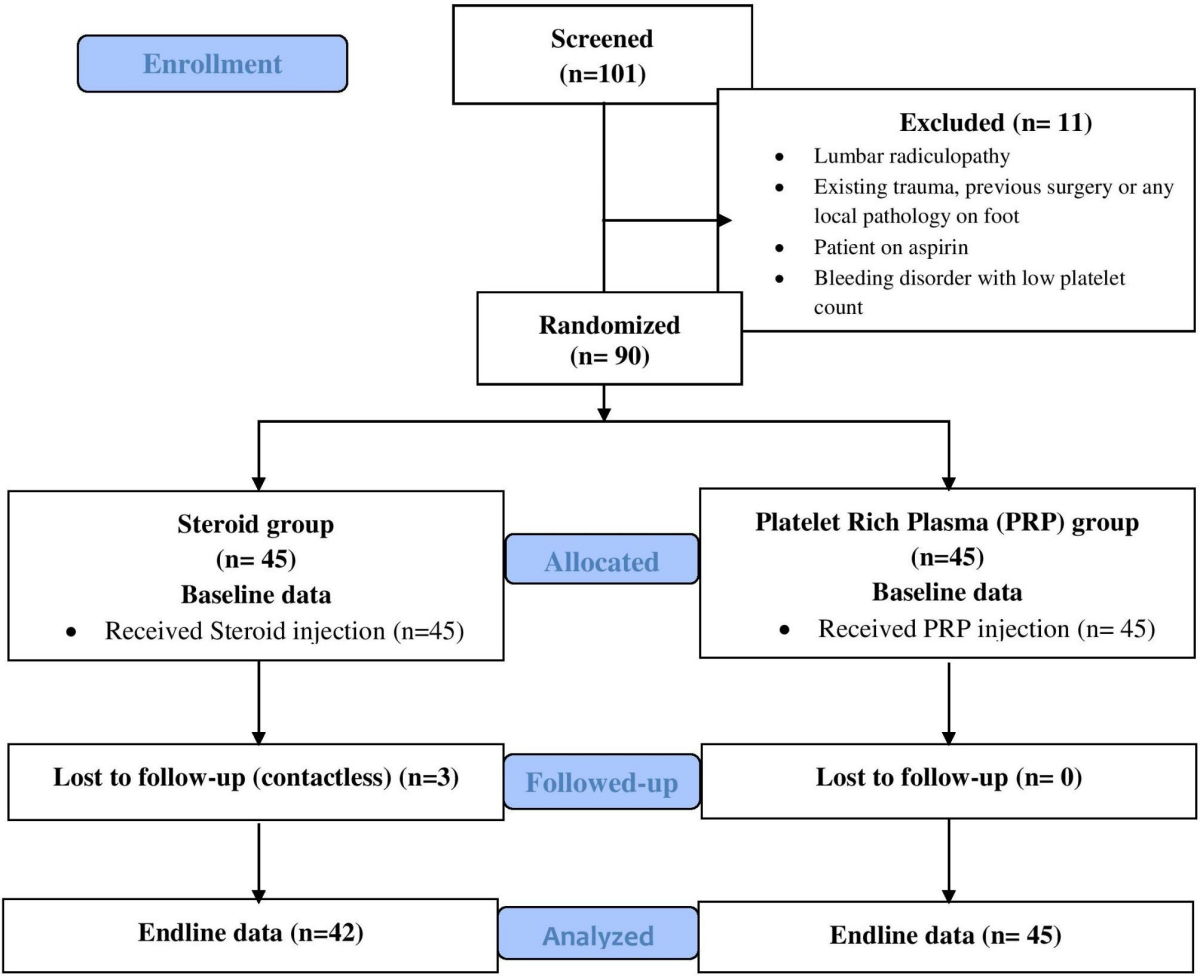
Flow chart of the study

After the assessment of the participants by the orthopaedic surgeon, they were randomized in a 1:1 ratio and assigned either to the steroid group or the PRP groups through computer-generated random numbers [61] (Fig. 1). A statistician prepared a computer-generated random number list allocated to the steroid group and PRP group. The allocated numbers were put inside the envelope by the statistician. The data enumerator opened the sealed envelope consecutively, with no exception. According to the information provided inside the envelope, participants were allocated either to the steroid group or the PRP group. Upon confirmation of a participant’s eligibility, the next envelope in the sequence was opened, and the treatment allocation was entered on a randomization list. A similar procedure of randomization technique was used in the previous study [62].

### Intervention

The patients diagnosed with plantar fasciitis by clinicians were randomized either into the steroid group or PRP group by the principal author and intervened by other research team members.

### Steroid group

In the steroid group, 2ml of injection Depo-Medrol 80 mg (Methylprednisolone) along with 1 ml lignocaine (0.25%) were loaded in a 5 cc syringe. Then the cocktail was injected into the medial calcaneal tuberosity at the most tender point using an aseptic technique [63].

### PRP group

The 30 ml blood of participants was collected into an acid citrate dextrose tube under aseptic conditions and subjected to centrifugation at 2000 rpm (soft spin) through a digital centrifuge machine speed control (REMI, R-8 C PLUS). There were three layers of blood; among them, the supernatant layer and buff coat of plasma were again subjected to centrifuge at 3000 rpm (hard spin). The upper two-thirds of the tube containing platelet-poor plasma was discarded, and the lower one-third of concentrated platelet plasma superficial buffy coat was injected into medial calcaneal tuberosity at the most tender point. The PRP preparation method and the way of injection technique were adapted from the previous study [64].

After the injection in both groups, the participants were advised not to engage in any rigorous activity with the affected foot for at least two days and then gradually return to their regular activities. All participants were counselled to follow up in the next visit at three months and six months. The midline and end-line data were recorded at three and six months, respectively.

### Study variables

#### Outcome variables

The primary outcomes were the functional mobility and pain of the participants; the secondary outcome measure was the plantar fascia thickness. The American Orthopaedic Foot & Ankle Society (AOFAS) [65] was used for the evaluation of functional mobility in the clinical setting before intervention (baseline) and after intervention (midline) at three months and (end-line) at six months follow-up. It combined subjective scores of pain and function provided by the patient as well as objective scores evaluated by the orthopaedic surgeon with a physical examination of the participants. They were assessed by sagittal motion, hindfoot motion, ankle–hindfoot stability, and alignment of the ankle–hindfoot. The AOFAS included nine items divided into three subscales (pain, function, and alignment). Pain consists of one item with a maximal score of 40 points, indicating no pain; function consists of seven items with a maximal score of 50 points, indicating full function and alignment consists of one item with a maximal score of 10 points, indicating good alignment. The maximal score is 100 points, indicating no symptoms or impairments [65]. Like AOFAS, the pain was evaluated at baseline and the midline at three months and end-line at six months with the Visual Analog Scale (VAS) [66]. The VAS consisted of a 10 cm straight line with the endpoints defining the intensity of pain from zero to 10 indicating that zero indicated “no pain at all” and 10 indicated “worst pain”. They were asked to express their severity of pain at the time of data collection in a descriptive term as no pain, mild pain, moderate pain, severe pain, or worst pain, and they were ranked to numerical scores for analysis [62]. Another outcome was the plantar fascia thickness which was assessed before intervention (baseline) and after intervention at six months through high-resolution ultrasonography (Ultrasound machine-SONOACE X7) by a trained radiologist. The cut-off value for confirming PF was used if plantar fascia thickness was more than 4 mm [67].

#### Predictor variables

Socio-demographic information of the respondents, such as age, sex, ethnicity, and religion, was collected using a semi-structured questionnaire through a face-to-face interview. Similarly, a clinical parameter such as direct radiographs of the heel was performed to find out the presence/absence of calcaneum spur and other heel pathologies.

### Safety issue

Most of the complications related to these interventions were analyzed. The side effect of the steroid varies based on the body site where it is injected; in the joint, muscle or spine. A review done by Hynes and Kavanagh, 2022 reveals that extra-articular steroid injection reports minor and major events in 0.81% and 0.5 to 8%, respectively while the injection in shoulder joints presents the major reaction in 18.1% [68]. As in our study, the injection was locally applied to the heel region; there were few chances of side effects like pain and discomfort for a few days, temporary bursitis, and flushing of the face for a few hours. The systemic side effects of local steroid injection are poorly understood and not well recognized, hence clinically insignificant [69]. Although there is a rise in blood glucose in diabetic participants, it is considered clinically insignificant [70, 71]. Plantar fascia rupture and heel fat pad atrophy are associated with local steroid injections in the long term which is around only 2.4–6.7% [72]. The steroid injection may develop temporary or permanent neural dysfunction leading to economic or social disabilities [73]. Hypopigmentation and atrophy of the skin may occur [74, 75], which is interestingly re-pigmented with exposure to ultraviolet light after a few months [76, 77]. Moreover, normal saline injection is considered a very effective modality to treat progressive cutaneous atrophy [78]. However, there were no such cases in our study. On the other side, PRP treatment is considered a safe and effective approach having very less side effects [33]. As this study was performed in a highly specialized tertiary hospital, the institution had a well-managed setup to handle in case of any immediate adverse reaction occurred.

### Ethical consideration

Ethical clearance was obtained from the Ethical Review Board (ERB) of the Nepal Health Research Council (NHRC) (Ref. 3322). Similarly, the clinical trial was registered to clinicaltrilregistry.gov (Identifier: NCT04985396, registered on 02/08/2021). Formal permission was obtained from the Nepal Orthopaedic Hospital, Kathmandu, Nepal to conduct the study at their site. We constituted a Data and Safety Monitoring Board (DSMB) consisting of two orthopaedic surgeons and one statistician. The DSMB members prepared study-stopping rules and reviewed all the possible effects reported. The respondents were informed about the purpose of this study. The signing of informed consent was taken from eligible participants. Voluntary informed participation and freedom of refusal at any time during the study were strongly applied so that participants could withdraw from the study at any time without giving a reason and without fear. Privacy and confidentiality of collected information were ensured at all levels.

### Data management and analysis

The collected data were entered in Epi-Data version 3.2 and analyzed based on the intention-to-treat (ITT) principle using Stata/MP version 14.1 (StataCorp LP, College Station, Texas). The normality of data was assessed using Shapiro–Wilk test. Socio-demographic data were analyzed using descriptive analysis. Since the data were normally distributed, the mean and standard deviation (SD) were calculated. Comparisons were made using the Student’s two-sample t-test. The Box and whisker plot was also used to display the PRP and steroid group outcome measures. All values less than 0.05 were considered statistically significant.

## Results

### Socio-demographic characteristics and clinical parameters of participants

At baseline, a total of 90 participants were included in this study (45 participants in the PRP and 45 in the steroid group). However, after six months of follow-up, a total of 87 participants completed the study (45 participants in the PRP group and 42 participants in the steroid group), whereas three participants lost follow in the steroid group, and none of the participants discontinued the trial in the PRP group (Fig. 1).

The majority, 76 (84.4%), were female, and 14 (15.6%) were male. The mean ± SD age of the participants was 43.8 ± 10.9 years (42.9 ± 10.3 in the PRP group and 44.7 ± 11.6 in the steroid group). Almost two-thirds of the participants (75.6%) were *Hindu* believers, while the remaining (24.4%) were of non-*Hindu* origin. Approximately equal proportions (48.9% and 51.1%) were from advantaged and disadvantaged ethnic groups, respectively. Most of the participants (58.9%) had the problem of plantar fasciitis on the right foot, and only 41.1% had the occurrence on the left side. Similarly, the presence of calcaneum spur was found in 52.2% of participants, while it was absent in 47.8% of participants **(**Table 1**)**.

**Table 1:** Demographic characteristics and clinical parameters of participants

Variables	Total (n = 90)	PRP treated group (n = 45)	Steroid treated group (n = 45)
	n (%)	n (%)	n (%)
**Sex**			
Female	76 (84.4)	39 (51.3)	37 (48.7)
Male	14 (15.6)	6 (42.8)	8 (57.2)
**Age categories**			
18–40	35 (40.2)	20 (54.1)	15 (45.9)
41–60	52 (59.8)	25 (47.2)	27 (52.8)
**Age in years (mean** **±** **SD)**	43.8 ± 10.9	42.9 ± 10.3	44.7 ± 11.6
**Ethnicity**			
Advantaged ethnic group	44 (48.9)	23 (51.3)	21 (48.7)
Disadvantaged ethnic group	46 (51.1)	22 (42.8)	24 (57.1)
**Religion**			
Hindu	68 (75.6)	35 (51.5)	33 (48.5)
Non-Hindu	22 (24.4)	10 (45.5)	12 (54.5)
**Clinical parameters**			
**Side**			
Left	37 (41.1)	18 (48.6)	19 (51.3)
Right	53 (58.9)	27 (50.9)	26 (49.1)
**Presence of calcaneum spurs**			
No	43 (47.8)	24 (55.8)	19 (44.2)
Yes	47 (52.2)	21 (44.7)	26 (55.3)

### Primary outcomes in steroid injection and PRP injection

The mean ± SD VAS scores for pain at baseline were 5.22 ± 1.34 and 4.77 ± 0.95 in PRP and SI groups, respectively with a group difference of 0.44 (95% CI: -0.04 to 0.93). The mean baseline pain scores were changed to 4.22 ± 1.04 in the PRP group and 3.14 ± 0.81 in the SI group in three months follow-ups with a group difference of 1.07 (95% CI: 0.67 to 1.47). Similarly, the baseline pain score was significantly decreased in the PRP group than the SI group (1.97 ± 1.13 versus 2.71 ± 0.94) with a group difference of -0.73 (95% CI: -1.18 to -0.28) in six months follow-up.

**Fig. 2:**
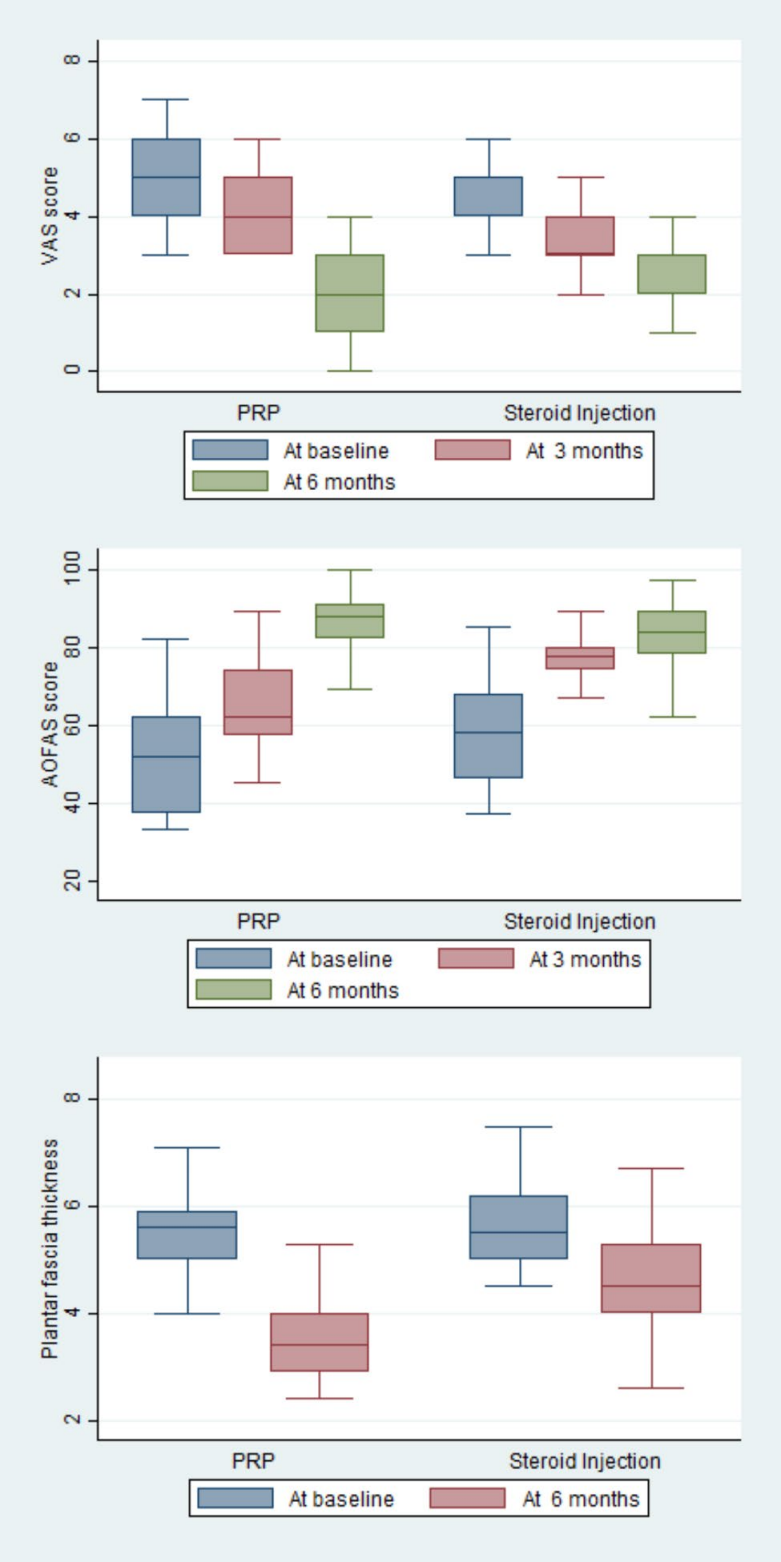
Box and whisker plot showing the comparison of different outcome variables between PRP and steroid groups

The functional mobility measured with the AOFAS scores were 52.53 ± 14.87 and 58.14 ± 11.47 in PRP and SI groups, respectively with a difference of 0.44 (95% CI: -0.04 to 0.93) at the baseline study. The mean AOFAS score was improved to 63.80 ± 12.04 in the PRP group and 75.76 ± 7.18 in the SI group in three months with a group difference of -11.96 (95% CI: -16.22 to -7.69). Similarly, the AOFAS score was significantly increased in PRP than SI group (86.04 ± 7.45 versus 81.23 ± 9.60) with a group difference of 4.80 (95% CI: 1.15 to 8.45) in six months (Table 2; Fig. 2).

### Secondary outcomes

The plantar fascia thickness between PRP and steroid groups was comparable (5.56 ± 0.95 mm versus 5.69 ± 0.88 mm) with the group difference of -0.12 (95% CI: -0.51 to 0.25) at baseline data which was decreased to 3.53 ± 0.81 mm and 4.58 ± 1.02 mm in six months, respectively with a difference of -1.04 (95% CI: -1.44 to -0.65) (Table 2; Fig. 2).

**Table 2:** Comparison of VAS score, AOFAS score, and plantar fascia thickness from baseline, three months to end line between the PRP treated and steroid groups

Variables	PRP treated group (n = 45)	Steroid treated group (n = 42)	Between-group difference	p-value^†^
	Mean (SD)	Mean (SD)	(95% CI)	
**VAS score**				
** At baseline**	5.22 (1.34)	4.77 (0.95)	0.44 (-0.04 to 0.93)	0.073
** At 3 months**	4.22 (1.04)	3.14 (0.81)	1.07 (0.67 to 1.47)	< 0.001*
** At 6 months**	1.97 (1.13)	2.71 (0.94)	-0.73 (-1.18 to -0.28)	0.001*
**AOFAS score**				
** At baseline**	52.53 (14.87)	58.14 (11.47)	-5.60 (-11.30 to 0.08)	0.005
** At 3 months**	63.80 (12.04)	75.76 (7.18)	-11.96 (-16.22 to -7.69)	< 0.001*
** At 6 months**	86.04 (7.45)	81.23 (9.60)	4.80 (1.15 to 8.45)	0.011*
**Plantar fascia thickness**				
** At baseline**	5.56 (0.95)	5.69 (0.88)	-0.12 (-0.51 to 0.25)	0.507
** At 6 months**	3.53 (0.81)	4.58 (1.02)	-1.04 (-1.44 to -0.65)	< 0.001*

^**†**^Student’s two-sample t-test; *Statistically significant at p < 0.05

## Discussion

The study aimed to compare the effect of PRP injection with steroid injection for the treatment of plantar fasciitis. This study shows that steroids had better results than PRP in three months, but in six months, PRP decreased the massive pain and had a more improved AOFAS score compared with steroids.

The well-being of the participants, assessed in terms of pain and functional mobility, was found to be better in the steroid group at three months; however, long-lasting relief from pain and higher mobility function was achieved at six months in the PRP group. These findings are consistent with other studies [60, 79]. Different systematic reviews have shown that steroid injection had a quick recovery in reducing the symptoms than PRP, which has a slower improvement but long-term permanent effect [50, 80]. Yang et al., 2017 found that the PRP is better than steroid injection for long-term pain reduction in plantar fasciitis, but there was no noticeable observed field difference between short- and intermediate-term effects [51]. This can be explained by the fact that PRP has growth factors and many other molecules with biological regenerative properties for the healing [81]. About 70% of growth factors are released after 10 min of PRP injection within one hour, which synthesize and secrete further growth factors for about eight days until the platelets die. It needs six to eight weeks for full activities after injection [82]. Steroids lack this property and interrupt the inflammatory and immune cascade, which is short-lived [80, 83]. Ang et al., 2019 found in the context of lateral epicondylitis that corticosteroid relieves acute pain but not in the long term, which may be due to the short half-life of the steroid [37]. It might be the reason that local steroid leads to a quick recovery in patients. So, they resume injurious activity without proper rehabilitation, which may lead to recurrence at a higher rate [27, 37]. Besides these, current knowledge reveals that PF occurs through a degenerative rather than an inflammatory process [84]. Histologically, PF has a small tear of fascia, which is replaced with normal fascia and surrounding tissue by angiofibroblastic hyperplastic tissue during the healing process. It is possible with the presence of anti-inflammatory and pro-inflammatory cytokines and interleukins, such as interleukin 4, 8, 13, interferon-α, and tumour necrosis factor-α in PRP [51]. Similarly, plantar fasciitis lacks the different growth factors due to hyper-vascularity and hypo-cellularity, and PRP provides these factors [85].

The findings of this study showed the comparable thickness of the plantar fascia in both PRP and steroid groups (5.56 ± 0.95 mm versus 5.69 ± 0.88 mm) at baseline which confirmed the plantar fasciitis; the cut-off value of more than 4 mm thickness of plantar fascia is suggested of plantar fasciitis [67, 86]. Our study found an immense reduction of plantar fascia thickness in the PRP group than in the steroid group in six months, which was clinically and statistically significant. Kalia et al., 2021 mention that steroid injection significantly reduces the plantar fascia thickness at one and three months than that of PRP but no difference in six months [87]. McMillan et al., 2012 explore that steroid injection reduces abnormal swelling of plantar fascia for up to three months [88]. Data reveals a 35.45% reduction in the thickness of plantar fascia in the PRP group and a 29.16% reduction in the corticosteroid group within six months of follow-up [85]. The mechanism of reducing the plantar fascia thickness due to the steroid and PRP injection has not been well explored. However, it can be justified through the fact that plantar fascia thickening is related to the inflammation episode [5], and both steroid and PRP have anti-inflammatory properties, and it reduces the inflammation [26, 89]. However, PRP may be advantageous over steroid injection as PRP may modulate the plantar fascia degeneration because of its regenerative properties, which steroid lacks and thus short-lived [26]. In fact, plantar fasciitis is degenerative pathology rather than a primarily inflammatory condition [90]. Steroid only reduces the pain temporarily but has no role in healing [91]. Unlike steroid, the effect of PRP does not wear off with time after six months [60]. The bioactive components of PRP help in tissue repair and wound healing by stimulating new blood cell formation and, thus, bringing nutrients and increased blood flow to the injury site [34]. The growth factors and cytokines present in PRP enhance the production of hyaluronan [92, 93], which is anti-inflammatory and increases the gliding between the deep fascia and muscle, reducing the plantar fascia thickness [6]. Furthermore, PRP contains a higher concentration of platelet [94], and higher percentages of lymphocytes and monocytes than whole blood, enhancing safe and natural healing [95] and thus, considered as an alternative to surgery [52–56]. Bohlen et al., 2020 conclude that PRP therapy has clinically similar outcomes compared to those of surgery in the treatment of type 1 medial epicondylitis [52]. Besides these, the success rate of surgical release accounts for 70–90%, with the complications of flattening the longitudinal arch at more than 50% complication rate and hypoesthesia [57]. On subjective evaluation, surgery presents about 80% of patients satisfied [57, 96], while PRP injection has 64% patient satisfaction [97].

This study has a few limitations. As this study was conducted in a specialized orthopaedic hospital, most of the patients had a treatment done earlier in another center which might affect our intervention’s outcomes. Also, most of the patients with plantar fasciitis preferred conservative treatment over injection therapy which could not make the larger sample size to generalize the findings in a large population. Similarly, the multivariate analysis could not be applied as we had no confounders of the plantar fasciitis. Besides these, we did not collect data on anthropometric measurements to find out the body mass index, which is associated with the mechanical properties of the plantar fascia and heel pad [98]. Similarly, we could not assess the participants’ plantar fascia thickness for three months to observe the pattern of changes in the thickness due to high cost. In addition, the data collection time has coincided with the COVID-19 pandemic, which distracted the data collection process due to the worrying scenario and lack of communication with participants. We developed a rapport with participants through compassionate empathy for the initiation of information sharing in data collection. Despite these few limitations, our study has some strengths too. First, we measured the plantar fascia thickness in six months follow-up, which was not found in any other previous studies. To the best of our knowledge, this might be the first study to evaluate the effect of PRP injection compared with SI in terms of the plantar fascia thickness. Second, we prepared PRP in the laboratory through double spinning centrifugation methods, which have a uniform and high concentration of platelet in the PRP. The commercially available PRP kits have a high cost, limited volume of drawn blood, and wide variation in platelet concentration [38]. Manually prepared PRP with double spin methods has high platelet capture efficiency (mean 47.11%, median 41.75%) than commercially available kits (mean 31.89%, median 29.51%) [99].

## Conclusion

The PRP injection showed better performance than the steroid injection for the treatment of plantar fasciitis in six months. To generate robust evidence comparing the efficacy of PRP to steroid injection for the treatment of plantar fasciitis, larger multi-centre trials with more than six months of follow-up are required.

## Electronic supplementary material

Below is the link to the electronic supplementary material.

Supplementary Material 1

## Data Availability

The data supporting this study’s findings are available on request from the corresponding author. The data are not publicly available due to privacy or ethical restriction.

## Abbreviations

AOFAS: American Orthopaedic Foot and Ankle Society
DSMB: Data and Safety Monitoring Board
ERB: Ethical Review Board
NHRC: Nepal Health Research Council
NSAIDs: Non-Steroid Anti-inflammatory Drugs
OPD: Outpatient Department
PF: Plantar Fasciitis
PRP: Platelet Rich Plasma
SI: Steroid Injection
VAS: Visual Analogue Score
