# Supplementary material for: Effect of platelet-rich plasma versus steroid injection in plantar fasciitis: a randomized clinical trial
Source: BMC Musculoskelet Disord. 2023 Mar 7;24:172. doi: 10.1186/s12891-023-06277-1 (PMC9989576; doi:10.1186/s12891-023-06277-1)

**ANNEX-I**

**Participant information**

**Effect of Platelet Rich Plasma versus Steroid injection in Plantar Fasciitis: A Randomized Clinical Trail**

**Introduction:**

Participant’s name (if agreed)……………………………….

**Participant ID Number……………………. Date (dd/mm/yy) ……………….**

**Mobile number…………………………………………………..**

**Patient’s consent: ……………….**

**Patient’s general information:**

| S.N. | Questions | Responses | Codes | Skip/Remarks |
| --- | --- | --- | --- | --- |

**Section 1: Socio-Demographic characteristics**

| 1. | Age | ………years |  |  |
| --- | --- | --- | --- | --- |
| 2. | What is your education level? | No education  Literate only  Primary (1-5)  Some secondary (6-9)  SLC or above | 1  2  3  4  5 |  |
| 3. | What is your caste/ethnicity? | Hi Hill Brahmin  Hi Hill Chhetri  Te Terai Brahmin/Cheetri  Other Terai Caste  Hi Hill Dalit  Te Terai Dalit  Ne Newar  Hi Hill Janjati  Other, Specify……………….. | 1  2  3  4  5  6  7  8  9 |  |
| 4. | What is your religion? | Hindu  Budhist  Muslim  Kirat  Christian  Others… | 1  2  3  4  5  6 |  |
| 5. | What is your occupation? | Housewife  Farmer  Employee  Trader  Unemployed | 1  2  3  4  5 |  |
| 6. | Ecological region | Mountain  Hill  Terai | 1  2  3 |  |
| 7. | Ecological region | Rural  Urban | 1  2 | VDC are rural, Municipality are urban |

**Section 2 clinical evaluation**

| 8. | Which side affected | Left  Right | 1  2 |  |
| --- | --- | --- | --- | --- |
| 9. | From when heel pain occur | ……..months |  |  |
| 10. | Other treatment done | No  Yes | 0  1 |  |

**Section 3: Anthropometric measurement**

| 11. | **Height (in cm)** | **Weight (in Kg)** | **BMI** |
| --- | --- | --- | --- |
|  |  |  |  |

**Section 4: Radiological evaluation**

| 12. | Calcaneum spur | No  Yes | 0  1 |
| --- | --- | --- | --- |
| 13. | Plantar fascia thickness | ……mm (Baseline)  ……mm (End line) |  |

**Annex-II**

**The American Orthopedic Foot and Ankle Society (AOFAS) Score:**

**Ⅰ Pain (40 points)i**

|  |  | **Baseline** | **End line** |
| --- | --- | --- | --- |
| None | 40 |  |  |
| Mild, occasional | 30 |  |  |
| Moderate, daily | 20 |  |  |
| Severe, almost always present | 0 |  |  |

**Ⅱ Function (50 points)**

| **Activity limitations, support requirement** |  |  |  |
| --- | --- | --- | --- |
| No limitations, no support | **10** |  |  |
| No limitation of daily activities, limitation of recreational activities, no support | **7** |  |  |
| Limited daily and recreational activities, cane | **4** |  |  |
| Severe limitation of daily and recreational activities, walker, crutches, wheelchair, brace | **0** |  |  |

| **Maximum walking distance, blocks** |  |  |  |
| --- | --- | --- | --- |
| Greater than 6 | 5 |  |  |
| 4-6 | 4 |  |  |
| 1-3 | 2 |  |  |
| Less than 1 | 0 |  |  |

| **Walking surfaces** |  |  |  |
| --- | --- | --- | --- |
| No difficulty on any surface | 5 |  |  |
| Some difficulty on uneven terrain, stairs, inclines, ladders | 3 |  |  |
| Severe difficulty on uneven terrain, tairs, inclines, ladders | 0 |  |  |

| Gait abnormality |  |  |  |
| --- | --- | --- | --- |
| None, slight | 8 |  |  |
| Obvious | 4 |  |  |
| Marked | 0 |  |  |

| **Sagittal motion (flexion plus extension)** |  |  |  |
| --- | --- | --- | --- |
| Normal or mild restriction (30° or more) | 8 |  |  |
| Moderate restriction (15°-29°) | 4 |  |  |
| Severe restriction (less than 150) | 0 |  |  |

| **Hindfoot motion (inversion plus eversion)** |  |  |  |
| --- | --- | --- | --- |
| Normal or mild restriction (75%-100% normal) | 6 |  |  |
| Moderate restriction (25%-74% normal) | 3 |  |  |
| Marked restriction (less than 25% normal) | 0 |  |  |

| **Ankle-hindfoot stability (anteroposterior, varus-valgus)** |  |  |  |
| --- | --- | --- | --- |
| Stable | 8 |  |  |
| Definitely unstable | 0 |  |  |

**III. Allignment- 10 points**

| Good, plantigrade foot, midfoot well aligned | 10 |  |  |
| --- | --- | --- | --- |
| Fair, plantigrade foot, some degree of midfoot malallgnment observed, no symptoms | 5 |  |  |
| Poor, nonplantigrade foot, severe malallgnment , symptoms | 0 |  |  |

**IV. Total Score (100 points):**

----------------- Pain Points +

-----------------Function Points +

----------------- Alignment Points =

**-----------------Total Points/100 points**

**Annex-III**

**Visual Analog Scale (VAS) Score:**


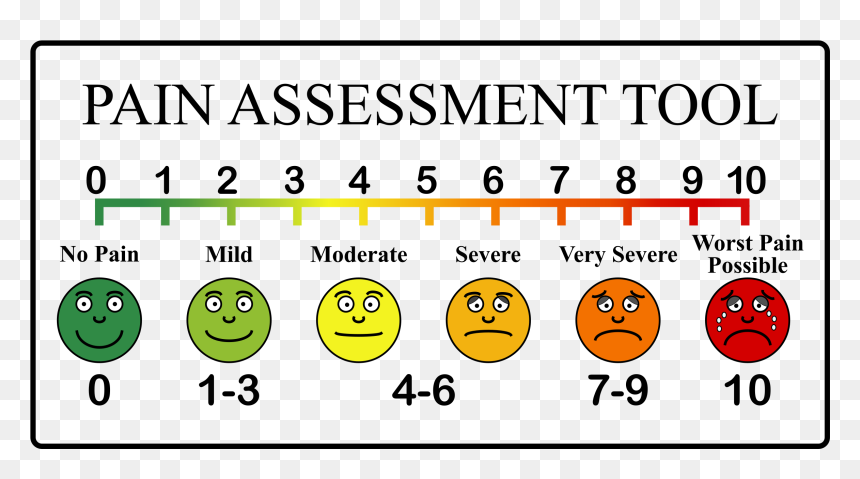

Supplement: Supplementary file 1 — Supplementary Material 1 [file 12891_2023_6277_MOESM1_ESM.docx]
